# Parvovirus B19 Uncoating Occurs in the Cytoplasm without Capsid Disassembly and It Is Facilitated by Depletion of Capsid-Associated Divalent Cations

**DOI:** 10.3390/v11050430

**Published:** 2019-05-10

**Authors:** Oliver Caliaro, Andrea Marti, Nico Ruprecht, Remo Leisi, Suriyasri Subramanian, Susan Hafenstein, Carlos Ros

**Affiliations:** 1 Department of Chemistry and Biochemistry, University of Bern, Freiestrasse 3, 3012 Bern, Switzerland; oliver.caliaro@dcb.unibe.ch (O.C.); andrea.marti@students.unibe.ch (A.M.); nicoolivier.ruprecht@insel.ch (N.R.); remo.leisi@dcb.unibe.ch (R.L.); 2 Department of Medicine, Pennsylvania State University College of Medicine, Hershey, PA 17033, USA; sxs1161@psu.edu (S.S.); suh21@psu.edu (S.H.); 3 Department of Biochemistry and Molecular Biology, Pennsylvania State University, University Park, PA 16802, USA

**Keywords:** B19V, parvovirus, uncoating, divalent cations, capsid stability, genome externalization, trafficking, nuclear targeting

## Abstract

Human parvovirus B19 (B19V) traffics to the cell nucleus where it delivers the genome for replication. The intracellular compartment where uncoating takes place, the required capsid structural rearrangements and the cellular factors involved remain unknown. We explored conditions that trigger uncoating in vitro and found that prolonged exposure of capsids to chelating agents or to buffers with chelating properties induced a structural rearrangement at 4 °C resulting in capsids with lower density. These lighter particles remained intact but were unstable and short exposure to 37 °C or to a freeze-thaw cycle was sufficient to trigger DNA externalization without capsid disassembly. The rearrangement was not observed in the absence of chelating activity or in the presence of MgCl_2_ or CaCl_2_, suggesting that depletion of capsid-associated divalent cations facilitates uncoating. The presence of assembled capsids with externalized DNA was also detected during B19V entry in UT7/Epo cells. Following endosomal escape and prior to nuclear entry, a significant proportion of the incoming capsids rearranged and externalized the viral genome without capsid disassembly. The incoming capsids with accessible genomes accumulated in the nuclear fraction, a process that was prevented when endosomal escape or dynein function was disrupted. In their uncoated conformation, capsids immunoprecipitated from cytoplasmic or from nuclear fractions supported in vitro complementary-strand synthesis at 37 °C. This study reveals an uncoating strategy of B19V based on a limited capsid rearrangement prior to nuclear entry, a process that can be mimicked in vitro by depletion of divalent cations.

## 1. Introduction

Human parvovirus B19 (B19V) commonly causes a mild childhood disease known as *erythema infectiosum*, or fifth disease [1]. In adults, the virus can cause a range of clinical manifestations, and infection during pregnancy may result in *hydrops fetalis* and foetal death [2]. B19V is transmitted principally through the respiratory route and targets the bone marrow where it infects and kills erythroblast precursors. The single-stranded DNA genome of B19V is packaged into a small, nonenveloped, T = 1 icosahedral capsid consisting of 60 structural subunits, of which approximately 95% are VP2 (58 kDa) and 5% are VP1 (83 kDa). VP1 and VP2 are identical except for 227 additional amino acids at the VP1 N-terminal region, the so-called VP1 “unique region” (VP1u) [3].

Viral capsids assemble as highly stable structures to retain and protect the genome during their extracellular phase. However, they also have a built-in ability for disassembly when entering a new host cell. These apparently contradictory functions are possible because the robust protective capsids are metastable. They are conceived to rearrange upon specific cellular cues, adopting a sequence of structural configurations in a stepwise manner. Those configurations enable the intracellular transport of capsids and the release of the genome in the appropriate cell compartment for replication [4]. Viral capsids have evolved various strategies to balance their stability outside of the cell against their capacity to disassemble inside the cell. The switch between capsid stability and instability is mediated by specific cellular cues. Cellular receptors, attachment factors, proteases, kinases, ubiquitin or cellular motors among others facilitate virus uncoating by direct interaction with the capsid. A particular intracellular environment, such as the low endosomal pH, reducing conditions or low calcium concentrations may also provide cues for uncoating [5,6,7]. During cell entry, parvoviruses traffic through various cellular compartments before they reach the cell nucleus where the viral genome is delivered for replication [8]. The intracellular compartment where uncoating takes place, the required capsid structural rearrangements and the cellular cues involved in the process are poorly understood.

Similar to other parvoviruses, B19V enters the cell through clathrin-mediated endocytosis [9]. Although the endocytic elements involved and the sites of escape into the cytosol may vary among parvovirus species and cells [10,11], parvoviruses depend on the endosomal acidification, notably to trigger the exposure of VP1u and its constitutive phospholipase A_2_ (PLA_2_) activity, required to promote endosomal escape [12]. In contrast to other parvoviruses, B19V does not require endosomal acidification for VP1u exposure, which occurs already at the cell surface to promote virus uptake [13,14,15,16]. However, low pH is still required for efficient endosomal escape. Accordingly, bafilomycin A_1_, which elevates the endosomal pH, but without compromising the integrity of endosomes, blocks the virus inside endocytic vesicles. In contrast, chloroquine, which induces endosomal vesicle enlargement and weakening, preventing their fusion to lysosomes [17], assists B19V infection by promoting endosomal escape [9]. The steps following the escape from endosomes are less well understood. Several studies have shown that cytoplasmic trafficking of parvovirus capsids is a microtubule-dependent process using cellular dynein as a motor protein [18,19]. However, other studies have shown that intracellular trafficking does not depend on dynein function or an intact microtubule network [20,21]. It has been proposed that parvoviruses enter the nucleus through the nuclear pore complex (NPC) via nuclear localization signals in the exposed VP1u [22,23,24,25,26]. A radically different mechanism has been suggested, which involves translocation of the capsids through discrete transient nuclear envelope (NE) breaks involving cell host caspases [27,28]. Through the NPC or through NE breaks, parvoviruses are small enough to enter the nucleus without capsid disassembly. However, it remains a matter of debate whether the infectious nuclear entry may still involve or not a disassembly process. Adeno-associated virus (AAV) infectivity can be blocked by injecting a neutralizing antibody against intact capsids into the nucleus [23]. However, other authors have shown that viruses enter the nucleus after partial or total disassembly in the cytosol or NPC [29]. 

A narrow channel at each five-fold vertex connecting with the interior of the particle is a common parvovirus structural feature and has been implicated in genome externalization and packaging [30,31,32,33,34]. In B19V capsids, the external end of the channel is closed, however, the presence of three consecutive glycine residues may provide the required flexibility to open the channel to allow the release of the viral DNA [35]. In agreement with this concept, in vitro studies have shown that parvovirus capsid disassembly is not required to externalize the viral genome [36,37,38,39,40,41]. Studies to understand the conditions required for DNA release at physiological conditions have shown that depletion of capsid-associated divalent cations in minute virus of mice (MVM) rendered the virions unstable and exposure to 37 °C was sufficient to trigger the externalization of the genome, which remained associated with the assembled capsid. The pressure of the encapsidated full-length viral DNA was important to promote the externalization at physiological temperatures [42]. A similar phenomenon was also observed in AAV, where the stability of virions containing full-length genomes required the presence of divalent cations in contrast to those containing subgenomic DNA [37,40].

Although the in vitro studies provide useful information, it remains uncertain whether the observed genome externalization without capsid disassembly can also occur in vivo during the process of virus entry. With the aim to gain insight into the mechanisms of B19V uncoating, conditions triggering viral DNA externalization at physiological temperatures were explored. To this end, virion density, capsid integrity and DNA accessibility were thoroughly examined in vitro upon exposure of virions to different conditions. Capsid rearrangements and uncoating were also followed in vivo during the process of cell entry. Viruses associated to cytoplasmic or to nuclear fractions were characterized at increasing times post-internalization by antibodies targeting capsid epitopes and phosphorylated amino acids. Additionally, the accessibility of the viral DNA was examined by immunoprecipitation and nuclease digestion and its suitability as a template for complementary-strand synthesis was evaluated by a primer hybridization and extension assay.

## 2. Materials and Methods

### 2.1. Cells and Viruses

The human megakaryoblastoid cell line UT7/Epo was cultured in Eagle’s minimal essential medium (MEM), supplemented with 5% fetal calf serum (FCS), 2 U/mL recombinant human erythropoietin (Epo) and penicillin/streptomycin. A B19V-infected plasma sample with a high viral load (genotype 1) was obtained from CSL Behring (Bern, Switzerland). The virus was concentrated by ultracentrifugation through 20% sucrose cushion. The virus pellet was resuspended in MEM, 20 mM HEPES or PBS and immediately used or stored at 4 °C.

### 2.2. Antibodies and Chemicals

The monoclonal antibody 860-55D (referred as Caps) was obtained from Mikrogen (Neuried, Germany). The antibody recognizes a conformational epitope expanding three neighboring VP2 molecules related by a five-fold and by a three-fold axis [43], and does not recognize disassembled capsids. This antibody was obtained from an infected healthy adult and is neutralizing [44]. The polyclonal rabbit antibody against the PLA_2_ region (referred as PLA2) was obtained as previously described [13]. The anti-phosphotyrosine mAb (clone 4G10), lambda phosphatase and the cytoplasmic dynein inhibitor ciliobrevin D (CbD) were purchased from EMD Millipore (Billerica, MA, USA). Anti-lamin A/C and anti-SERCA2 ATPase antibodies were obtained from Abcam (Cambridge, MA, USA). Bafilomycin A_1_ (BafA_1_) and chloroquine, were purchased from Sigma (St. Louis, MO, USA). CbD and BafA_1_ were solved in DMSO and CQ was solved in water.

### 2.3. Quantitative PCR

Amplification of B19V DNA and real-time detection of PCR products were performed using a CFX96 Real Time Detection System (Bio-Rad, Hercules, CA, USA). Quantitative PCR (qPCR) was performed using the iTaq^TM^ SYBR^®^ Green Supermix kit (Bio-Rad) following the manufacturer’s instructions. Primers used for B19V DNA amplification were as follows: B19V-forward, 5′-GGGCAGCCATTTTAAGTGTTT-3′; and B19V-reverse, 5′-GCACCACCAGTTATCGTTAGC-3′. Plasmids containing the complete genome of B19V were used at 10-fold dilutions as external standards.

### 2.4. Iodixanol Density Gradient Ultracentrifugation

The virus suspension was applied onto an iodixanol gradient containing 1.5 mL 55%, 2 mL 45%, 2 mL 40%, 2 mL 35% and 1.5 mL 15% iodixanol solution in 20 mM HEPES. The virus suspension was adjusted to 2 mL with the corresponding buffer and centrifuged at 35,000 rpm for 18 h at 18 °C in a swinging bucket rotor Beckman SW41Ti. After the centrifugation, 0.5 mL fractions were collected from the top. The refractive index was determined for each fraction and the presence of the virus was determined by dot-blot and qPCR.

### 2.5. Transmission Electron Microscopy

Purified B19V was resuspended in PBS alone or PBS supplemented with 1 mM MgCl_2_ and stored at 4 °C for one month. Following a freeze/thaw cycle, 3 µL of each sample was applied to a freshly glow-discharged continuous carbon grid from Electron Microscopy Sciences (Hatfield, PA, USA), washed three times with dH_2_O, and stained with 3 µL 1% (*w*/*v*) phosphotungstic acid. CCD images were acquired using a JEOL JEM 1200 EXII transmission electron microscope (Peabody, MA, USA) at 80 kV accelerating voltage, at 10,000× and 30,000× magnification.

### 2.6. Nuclease Assay

The presence of accessible DNA from the different virus samples was analysed by treatments with DNase I (Sigma). DNase I digestion was performed at 37 °C for 1 h in a buffer containing 40 mM Tris-HCl, pH7.9, 10 mM NaCl, 6 mM MgCl_2_ and 1 mM CaCl_2_. In order to test the DNase I activity, B19 viral particles were heated at 60 °C or 80 °C for 5 min. Native untreated virions served as controls. To stop the reaction, the viral DNA was extracted with the DNeasy blood and tissue kit (Qiagen, Venlo, Netherlands) and quantified as specified above.

### 2.7. Isolation of Cytoplasmic and Nuclear Fractions from Infected Cells

UT7/Epo cells (5 × 10^5^) were incubated with B19V (2 × 10^4^ virions per cell) for 1 h at 4 °C. The cells were subsequently washed four times at 4 °C with PBS, resuspended in MEM and further incubated at 37 °C to allow virus internalization. At increasing times post-internalization (pi), cytoplasmic or nuclear fractions were prepared. For cytoplasmic purification without nuclear contamination, cells were lysed in NP40 buffer (50 mM Tris-HCl, 150 mM NaCl, 1% NP-40) supplemented with a protease inhibitor cocktail (Complete Mini; Roche, Basel, Switzerland). After incubation on ice for 30 min, the cells were vortexed for 20 s and further incubated for 15 min on ice. Nuclei and cell debris were removed by centrifugation at 14,000× *g* for 10 min at 4 °C. The absence of nuclear contamination was determined by quantification of the human β-actin gene by qPCR.

For the isolation of nuclei, the cells were washed twice with ice-cold PBS and the pellets were resuspended in 100 µL EZ buffer (Sigma) and the volume completed to 1 mL with additional EZ buffer. The samples were vortexed and kept on ice for 5 min, then pelleted at 500× *g* for 5 min at 4 °C. This step was repeated once. Pellets were then resuspended in 500 µL EZ buffer containing 0.25 M sucrose and layered on top of 500 µL EZ buffer containing 0.5 M sucrose. The nuclei were collected by centrifugation at 500× *g* for 10 min at 4 °C, washed with 1 mL EZ buffer and resuspended in the desired buffer. The integrity of the isolated nuclei was assessed via light microscopy after trypan blue staining. The purity of the nuclei and the absence of cytoplasmic contamination were examined with antibodies against lamin A/C (marker for nuclear inner membrane), SERCA2 ATPase (marker for *endoplasmic reticulum*), and GAPDH (cytoplasmic marker). For immunoprecipitation, the nuclear pellets were resuspended in RIPA lysis buffer (50 mM Tris-HCl, 150 mM NaCl, 1% Triton X-100, 0.5% sodium deoxycholate, 0.1% SDS), supplemented with protease inhibitor cocktail (Complete Mini; Roche). The samples were incubated on ice for 20 min, vortexed for 20 s and further incubated for 15 min on ice. Nuclear debris was removed by centrifugation at 14,000× *g* for 10 min at 4 °C.

### 2.8. Immunoprecipitation

UT7/Epo cells were infected with B19V, as described above. At different times pi, viral particles were immunoprecipitated from cytoplasmic or from nuclear fractions with a B19V specific antibody against intact capsids (860-55D; Caps) or with an antibody against the PLA_2_ region in VP1u (PLA2). For immunoprecipitation of phosphorylated capsids, an antibody against phosphorylated tyrosine was used (pTyr). After overnight incubation with 20 μL protein G agarose beads in LoBind tubes (Eppendorf, Hamburg, Germany) at 4 °C, the beads were washed four times (three times with PBSA-1% bovine serum albumin and once with PBS) and resuspended in protein loading buffer to analyse the immunoprecipitated capsids by Western blotting or in PBS to quantify the viral DNA by qPCR. Total DNA was extracted using the DNeasy blood and tissue kit (Qiagen) and quantified as specified above. The immunoprecipitated capsids were also used for complementary-strand synthesis, as specified below. The antibody 860-55D (Caps) was also used in immunoprecipitation experiments and dot-blot assays to test the integrity of the capsids. 

### 2.9. Infectivity Assay

Virus infectivity was examined by quantification of NS1 mRNA. Cells were transferred 24 h pi to RNase-free tubes (Safe-Lock Tubes 1.5 mL, Eppendorf Biopur^®^) and pelleted. The pellet was washed twice with PBS and stored at −20 °C until use. Total poly-A-mRNA was isolated with a Dynabeads mRNA direct kit (Invitrogen). Following reverse transcription, cDNA was quantified by using iTaq^TM^ Universal SYBR^®^ Green One-Step reagent kit (Bio-Rad, Hercules, CA, USA). Primers were chosen to amplify a 133 nt-long NS1 cDNA fragment: NS1 forward (5’-GGGGCAGCATGTGTTAAAG-3’ (nucleotide 1017–1035) and NS1 reverse (5’-CCATGCCATATACTGGAACACT-3’ (nucleotide 1129–1150).

### 2.10. Complementary-Strand Synthesis

The presence of externalized DNA from the immunoprecipitated capsids and its suitability as a template for initiation of DNA synthesis was examined by a primer hybridization and extension reaction as previously described [38]. Briefly, primers consisting of a 3’ virus-specific and a 5’ virus-unrelated sequence were used. The hybridization reaction was performed in 40 μL volumes containing 20 μL of immunoprecipitated virus bound to protein G beads, 4 μL 5× hybridization buffer (40 mM Tris-HCl, pH 7.5, 20 mM MgCl_2_ and 50 mM NaCl) and 2 μL primer (0.5 pmol), at 37 °C for 15 min. The hybridized primer was extended by adding 2 μL DTT (100 mM), 2 μL dNTPs (200 μM each) and 4 μL (3.25 U) of T7 DNA polymerase (Sequenase; USB, Cleveland, OH, USA) and incubated at 37 °C for 15 min or 0 min (negative control). The reaction was stopped, and DNA was purified by using the Wizard^®^ SV Gel and PCR Clean-Up System (Promega, Madison, WI, USA). The primer-extended DNA was amplified by PCR as previously described [38] and the amplicons were separated by electrophoresis and visualized by staining with GelRed (Biotium, Hayward, CA, USA).

## 3. Results

### 3.1. Depletion of Capsid-Associated Divalent Cations Destabilizes the B19V Capsid

**Figure 1:**
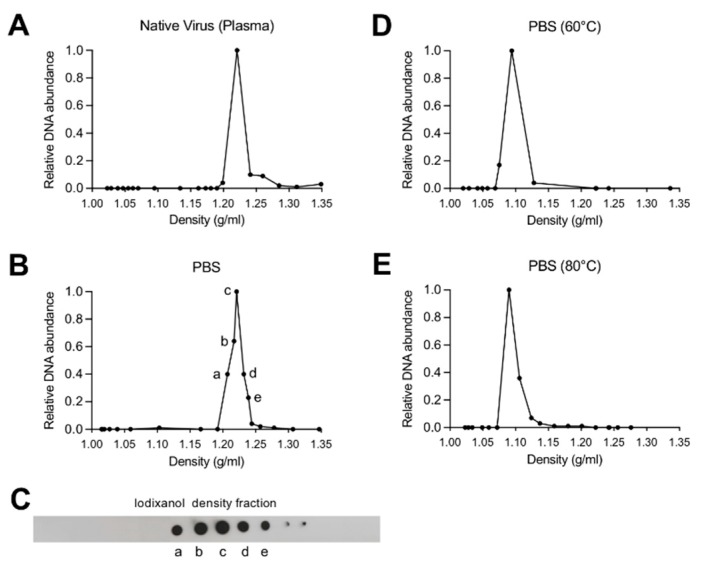
Buoyant density of native B19V from infected plasma or purified and resuspended in PBS. Fractions from iodixanol density gradient ultracentrifugation were collected and quantified by qPCR. (**A**) Density of B19V from infected human plasma. (**B**) Density of B19V purified from infected plasma by ultracentrifugation through 20% sucrose cushion and resuspended in PBS. (**C**) Detection of B19V capsids by dot-blot from density fractions of B19V in PBS. The corresponding fractions are indicated (a–e). (**D**) Density of B19V treated at 60 °C for 5 min. (**E**) Density of B19V treated at 80 °C for 5 min.

Iodixanol density gradient ultracentrifugation and qPCR were used to detect and to quantify changes in B19V capsid density. The density of native B19V from infected human plasma was compared to the virus pelleted through a 20% sucrose cushion and resuspended in PBS. Native capsids in human plasma and in PBS peaked at 1.22 g/mL, representing the density of intact native capsids (Figure 1A,B). A dot-blot confirmed the presence of capsids in the same fractions as the viral DNA (Figure 1C). Virus in PBS and exposed to 60 °C or 80 °C for 5 min resulted in a density shift to 1.09 g/mL, which corresponds to free viral DNA (Figure 1D,E).

**Figure 2:**
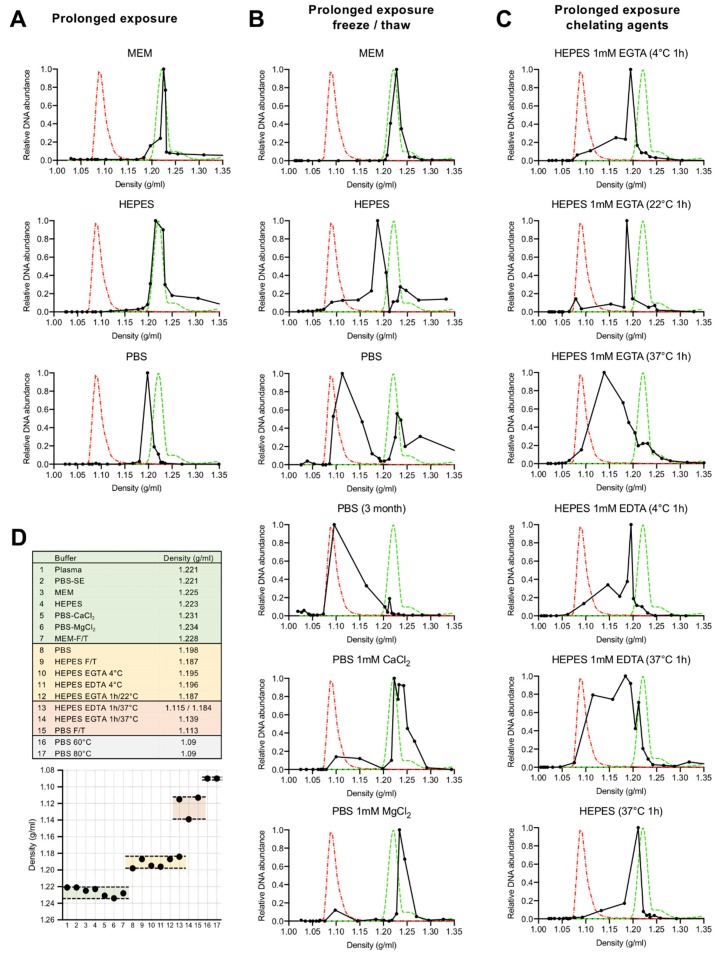
Influence of divalent cation depletion on the density of B19V. Virus from infected plasma was pelleted through 20% sucrose cushion, resuspended in different buffers and stored at 4 °C for one month. Fractions from iodixanol density gradient ultracentrifugation were quantified by qPCR. (**A**) Effect of prolonged (one month) incubation of B19V in MEM, HEPES and PBS at 4 °C. (**B**) Effect of a freeze/thaw cycle after prolonged incubation of B19V in MEM, HEPES, PBS and PBS supplemented with 1 mM CaCl_2_ or 1 mM MgCl_2_. (**C**) Effect of prolonged incubation of B19V in HEPES at 4 °C supplemented or not with 1 mM EGTA or 1 mM EDTA. Before separation by iodixanol density centrifugation, the virus suspensions were treated at 4 °C, 22 °C or 37 °C for 1 h. Green peak; density native virus. Red peak; density free viral DNA. (**D**) Upper panel; summary of the major density peaks (g/mL) of B19V in the different buffers and exposed to different conditions. Lower panel; graphic representation of the four distinct density groups. Similar densities appear grouped by colours.

The density of B19V resuspended in MEM, HEPES and PBS and stored for a period of four weeks at 4 °C (prolonged exposure) was compared. While the virus density in MEM and HEPES did not change (1.22 g/mL), prolonged exposure to PBS induced a structural rearrangement resulting in capsids with lower density (1.20 g/mL) (Figure 2A). We next analysed the stability of B19V after prolonged exposure (four weeks at 4 °C) to the different buffers followed by a single freeze/thaw cycle. While the virus in MEM did not change, the virus in HEPES peaked at a similar density as the virus that was exposed to PBS for a prolonged time at 4 °C (1.19 g/mL). In contrast, a major density shift was observed after a freeze/thaw cycle in the virus exposed to PBS (1.11 g/mL). This shift was fully prevented when PBS was supplemented with 1 mM of MgCl_2_ or 1 mM CaCl_2_ (1.23 g/mL), suggesting that divalent cations have a stabilizing effect on the virion (Figure 2B). To further study the influence of divalent cations, the effect of the chelating agents EDTA and EGTA on the virus density was analysed. Prolonged exposure (four weeks at 4 °C) to HEPES supplemented with 1 mM EGTA or 1 mM EDTA provoked a density shift to 1.195 g/mL, similar to the shift observed in the virus exposed to PBS. However, these lighter particles were unstable and short exposure to 37 °C was sufficient to trigger a major density shift, similar to that of virus in PBS and exposed to a freeze/thaw cycle. The rearrangement at 37 °C was not observed in the absence of chelating agents (Figure 2C).

In total, four density groups were identified (Figure 2D). Viruses in plasma or in buffers with divalent cations were stable and had a similar density around 1.22 g/mL. Viruses exposed to buffers with chelating activity peaked at a density of 1.19–1.20 g/mL. These particles shifted to 1.11–1.14 g/mL when exposed to 37 °C or to a freeze/thaw cycle. Free viral DNA generated by heat treatment of virions (60 °C or 80 °C for 5 min) peaked at 1.09 g/mL. 

### 3.2. Depletion of Divalent Cations Facilitates B19V Uncoating without Capsid Disassembly

**Figure 3:**
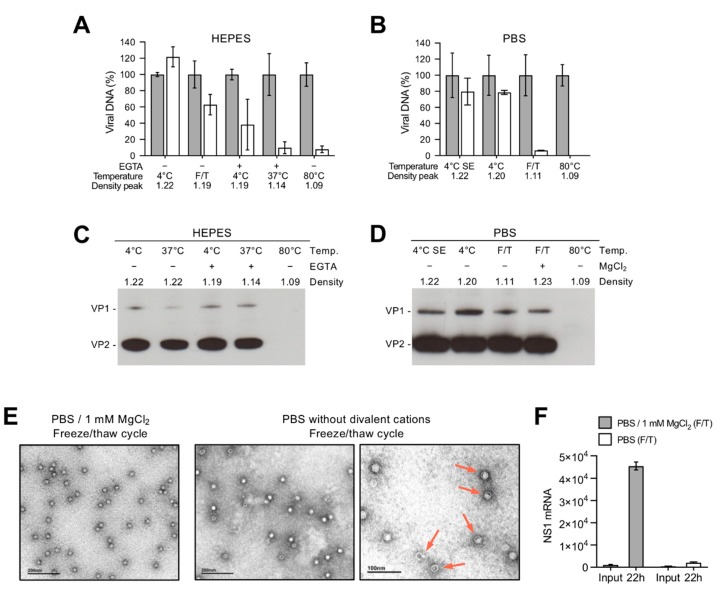
DNA accessibility and capsid integrity of the different B19V density populations. (**A**,**B**) Nuclease sensitivity of viruses in HEPES or in PBS displaying different densities. Virus samples from representative density populations were treated with DNase I at 37 °C for 1 h (white columns). Virus treated at 80 °C for 5 min was used as control. (**C**,**D**) Integrity of capsids in HEPES or in PBS displaying different densities. Virus samples from representative density populations were immunoprecipitated with the conformational antibody 860-55D against assembled capsids. Virus treated at 80 °C for 5 min was used as control. (**E**) Transmission electron microscopy (TEM) images of B19V in PBS alone and PBS supplemented with 1 mM MgCl_2_ and exposed to a freeze/thaw cycle. Arrows indicate capsids with rearranged DNA. (**F**) Infectivity assay based on NS1 mRNA detection with the same B19V preparations used for TEM. SE; short exposure. F/T; freeze and thaw. Values correspond to genome equivalents per microliter (geq/µL).

The capsid integrity of the different density populations was investigated by nuclease digestion and by immunoprecipitation with the antibody 860-55D against a conformational epitope (Caps), which recognizes only assembled capsids [44]. As shown in Figure 3A,B, capsids at densities ranging from 1.22–1.19 g/mL were mostly DNase I-resistant. Since the capsids were shortly exposed to 37 °C during the DNase I treatment, virions with densities around 1.19 g/mL showed a variable degree of nuclease sensitivity. However, capsids peaking at densities 1.11–1.14 g/mL were fully sensitive to nuclease digestion, similarly to control virus heated at 80 °C. The viral capsids could be immunoprecipitated under all tested conditions, except the control sample, which was exposed to 80 °C to provoke capsid disassembly (Figure 3C,D). This result indicates that the structural rearrangements resulting in DNA externalization do not compromise the integrity of the capsids, which remain assembled.

Negative stain transmission electron microscopy was used to visualize the effect on capsids and the packaged DNA in the absence of divalent cations. In PBS supplemented with 1 mM MgCl_2_ (Figure 2B; density 1.23 g/mL), the capsids appear as stable, DNA-filled capsids. In contrast, in the absence of MgCl_2_ and exposed to a freeze/thaw cycle (Figure 2B; major density peak at 1.11 and minor density peak at 1.22), a mixture of empty and DNA-filled capsids was observed. Some capsids showed an “eclipsing” effect, where the DNA appears condensed and rearranged, capturing an intermediate step during genome release (Figure 3E). The infectivity of the virus preparations used for TEM was evaluated by the quantification of the NS1 mRNA. While viruses supplemented with MgCl_2_ were infectious, the infectivity dropped significantly in the absence of divalent cations (Figure 3F).

In summary, four density populations were characterized and are outlined in Figure 2D. Viruses in plasma or in buffers with divalent cations were stable, nuclease resistant and had a similar density around 1.22 g/mL. Viruses exposed for a prolonged time to buffers with chelating activity or to chelating agents at low temperatures remained assembled but their density shifted to 1.19–1.20 g/mL. These particles were unstable and exposure to 37 °C or to a freeze/thaw cycle was sufficient to trigger the externalization of the viral DNA without capsid disassembly and to provoke a major density shift to 1.11–1.14 g/mL. Finally, heating to 60 °C or 80 °C for 5 min caused the complete release of the viral DNA, which peaked at 1.09 g/mL. These results together suggest that capsid-associated divalent cations have a stabilizing role and their removal prepares the virus for uncoating at physiological temperatures without capsid disassembly.

### 3.3. A Proportion of Incoming Capsids Uncoat in the Cytoplasm without Capsid Disassembly

**Figure 4:**
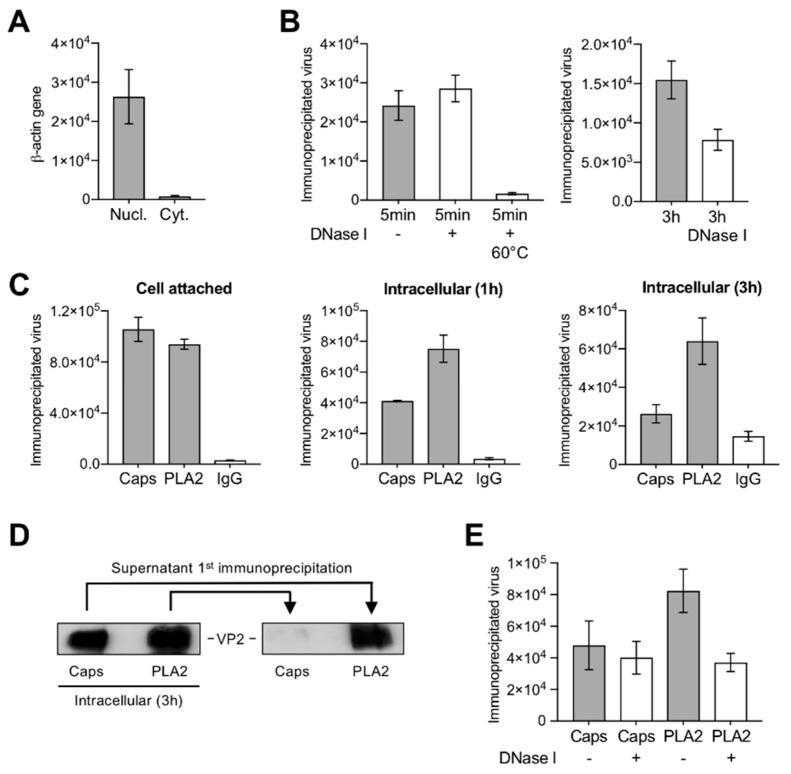
Capsid structural rearrangements and viral DNA externalization in the cytoplasm. (**A**) The purity of the cytoplasmic fraction and the absence of nuclear contamination were examined by quantitative detection of β-actin gene sequences. Values represent DNA copies per microliter. (**B**) Nuclease sensitivity of virions at 5 min and 3 h pi Virus treated at 60 °C for 5 min was used as control. (**C**) Accessibility of VP1u (PLA2 antibody) and VP2 conformational (Caps antibody) epitopes from membrane-bound or intracellular B19V. Virions were immunoprecipitated and quantified by qPCR. As control, an unrelated IgG was used. (**D**) Western blot of B19V immunoprecipitated at 3 h pi. The supernatants were used for a second immunoprecipitation with the indicated antibodies. (**E**) Nuclease sensitivity of B19V immunoprecipitated with the conformational antibody (Caps) or with the VP1u antibody (PLA2) at 1 h pi. All immunoprecipitation values represent geq/µL.

Cytoplasmic fractions were prepared from infected UT7/Epo cells. The lack of nuclear contamination was confirmed by quantitative detection of β-actin gene sequences (Figure 4A). The accessibility of the viral DNA from the incoming cytoplasmic virus was examined by nuclease digestion and qPCR. While at 5 min pi the viral DNA remained protected, approximately half of the incoming genomes became sensitive to nuclease digestion after 3 h pi (Figure 4B). The VP1u region is not accessible in native capsids but it becomes accessible upon interactions on the surface of susceptible cells [14,38]. As expected, cell-bound viruses were immunoprecipitated with the antibody targeting a conformational epitope in VP2 (Caps) and by the antibody targeting the PLA_2_ region in VP1u (PLA2). During cell entry, the PLA_2_ region remained accessible but the conformational epitope in VP2 became undetectable in an increasing proportion of incoming particles (Figure 4C). These results were further confirmed by immunoprecipitation and Western blot analysis of capsid proteins. Incoming viruses were immunoprecipitated with Caps and PLA2 antibodies at 3 h pi. The supernatant was used to immunoprecipitate remaining viruses with the heterologous antibody. While the PLA2 antibody detected all intracellular capsids, the Caps antibody did not recognize a significant proportion of incoming capsids (Figure 4D), suggesting that during entry some incoming virions rearrange and adopt a configuration that remained recognized by the PLA2 antibody but not by the VP2 conformational antibody.

We next verified whether the observed capsid structural rearrangement rendered the viral DNA accessible. To explore this possibility, virions from cytoplasmic fractions were immunoprecipitated with the Caps or with the PLA2 antibody 3 h pi and the accessibility of the viral DNA from the immunoprecipitated capsids was examined by nuclease digestion and qPCR. The incoming capsids that remained detectable by the conformational antibody were resistant to nuclease digestion. In contrast, approximately half of the virions that were immunoprecipitated with the PLA2 antibody were sensitive to nuclease digestion (Figure 4E). This result suggests that a significant proportion of incoming capsids uncoat in the cytoplasm through a limited capsid rearrangement.

### 3.4. Uncoating Occurs after Endosomal Escape

**Figure 5:**
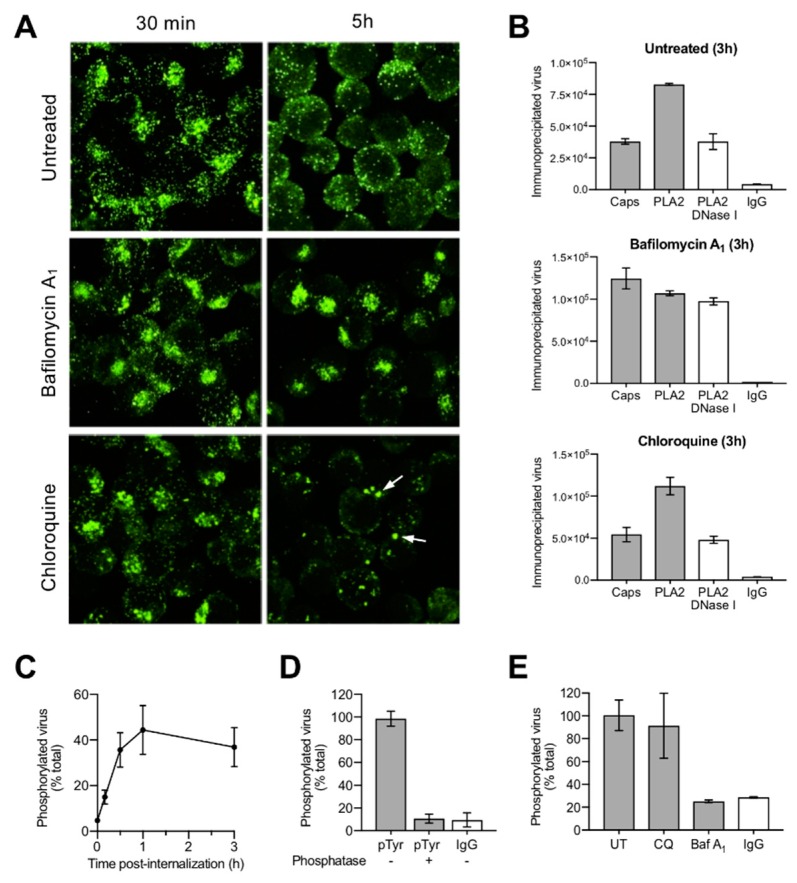
Conformational epitope rearrangement, DNA externalization and phosphorylation occur after endosomal escape. (**A**) Effect of BafA_1_ and CQ on endosomal escape and conformational epitope integrity. Immunofluorescence images of infected cells treated or not with BafA_1_ (20 nM) and CQ (25 µM). The integrity of the conformational epitope and the characteristic endocytic clustering of the virus was examined by staining with the conformational antibody (Caps) at 30 min and 3 h pi. Arrows indicate the presence of enlarged endosomes in cells treated with CQ. (**B**) Effect of BafA_1_ and CQ on the conformational rearrangement and nuclease sensitivity of the incoming virions. The virions were immunoprecipitated from infected cells at 3 h pi and quantified by qPCR, values correspond to geq/µL. (**C**) Kinetics of B19V phosphorylation. At increasing times pi, virions were immunoprecipitated from cytoplasmic fractions with PLA2 (total virions) or with an antibody against phosphotyrosine and quantified by qPCR. (**D**) Phosphatase treatment abrogated the reactivity with the phosphospecific antibody. Before immunoprecipitation, the intracellular capsids were treated or not with lambda phosphatase (500 U) for 1 h at 30 °C. (**E**) Effect of BafA_1_ (20 nM) or CQ (25 µM) on B19V phosphorylation. Virions from cytoplasmic fractions were immunoprecipitated with the phosphotyrosine antibody at 3h pi and quantified by qPCR. As control, an unrelated IgG was used.

In order to examine whether the detected capsid rearrangement and DNA externalization occur before or after endosomal escape, cells were treated with bafilomycin A_1_ (BafA_1_), which was previously shown to block endosomal escape of B19V [9]. The cells were fixed 30 min and 5 h pi and incoming viruses were detected with the conformational antibody (Caps). After 30 min, a similar signal with a characteristic endocytic distribution was observed in untreated or BafA1-treated cells. However, 5 h pi the number of capsids detectable with the conformational antibody decreased significantly in the untreated cells, but remained stable in the BafA_1_-treated cells and displayed the same endocytic distribution, indicating that endosomal escape and the capsid rearrangement were inhibited (Figure 5A). The capsid rearrangement and the accessibility of the DNA were also examined by immunoprecipitation and nuclease digestion. As shown in Figure 5B, while in untreated cells a proportion of incoming capsids rearranged and became nuclease sensitive, in the presence of BafA_1_ the capsids remained mostly unchanged.

These results are in line with our previous studies where we have shown that BafA_1_ inhibits B19V infection by blocking endosomal escape. In the same studies, we observed that chloroquine (CQ), which also raises endosomal pH, did not hinder endosomal escape [9], an effect that was attributed to the vacuolization and damage of endocytic vesicles [17]. Accordingly, CQ was used to verify whether the inhibitory effect of BafA1 on the rearrangement and DNA accessibility is due to the raise in the endosomal pH or to the block of endosomal escape. As shown in Figure 5A,B, CQ induced the enlargement of endocytic vesicles and did not prevent the capsid rearrangement and viral DNA externalization, suggesting that these changes ensue only after endosomal escape and are not mediated by the low endocytic pH.

### 3.5. Incoming Capsids Become Reactive to Phosphospecific Antibodies Following Endosomal Escape

Cellular kinases have been shown to phosphorylate incoming parvovirus capsids following endosomal escape [45]. We investigated whether B19V is also phosphorylated after endosomal escape. To this end, cell-bound and internalized capsids were incubated with a phosphospecific antibody targeting tyrosine phosphorylation. Quantification of the immunoprecipitated capsids by qPCR revealed that by 1 h pi, approximately half of the internalized capsids became phosphorylated (Figure 5C). Capsid phosphorylation initiated as early as 10 min pi and was maximal between 30 min and 1 h pi. Incoming B19V were not reactive to phosphospecific antibodies following incubation with lambda phosphatase (Figure 5D). As expected, inhibition of endosomal escape by BafA_1_ abrogated capsid phosphorylation. In contrast, phosphorylation was not prevented in the presence of CQ (Figure 5E), confirming our previous observations indicating that CQ does not hinder endosomal escape [9]. These results further demonstrate that the observed capsid rearrangement and DNA accessibility are not triggered by the endosomal low pH but following the delivery of incoming particles into the cytosol.

### 3.6. Capsids Phosphorylated and with Accessible Genomes Accumulated Progressively in the Nuclear Fraction

**Figure 6:**
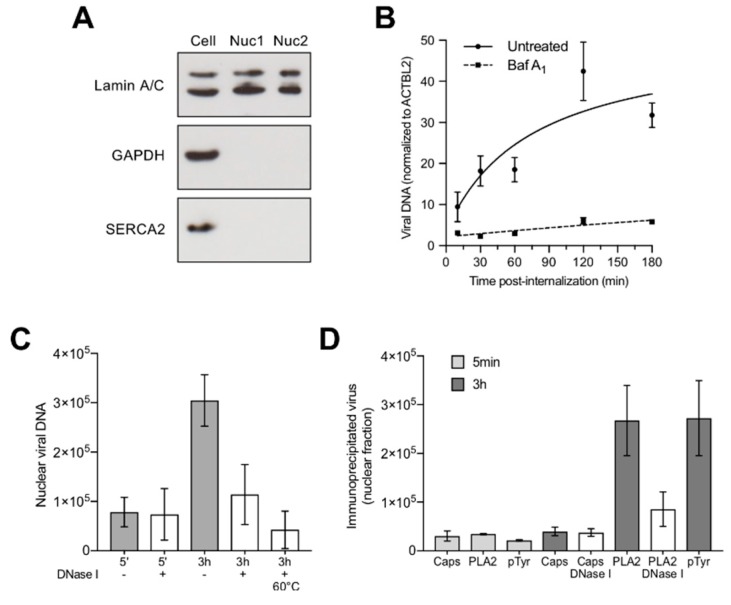
Characterization of incoming B19V associated to the nuclear fraction. (**A**) Isolation of intact nuclei without cytoplasmic contamination. The purity of the nuclear fraction and the absence of cytoplasmic contamination were examined by the detection of lamin A/C (nuclear marker) and SERCA2 ATPase (ER marker) and GAPDH (cytoplasmic marker) by Western blot. Nuc1 and Nuc2; two independent nuclear preparations. (**B**) Accumulation of B19V in the nuclear fraction at increasing times pi. Nuclei isolated after 5 min or from cells treated with BafA_1_ served as negative controls. The results were normalized by the quantification of the β-actin-like gene 2 (ACTBL2). (**C**) Nuclease sensitivity of virions associated to the nuclear fraction. Nuclei isolated after 5 min served as background control. Nuclear fractions prepared 3 h pi were treated at 60 °C for 5 min to control the activity of DNase I. qPCR values represent geq/µL. (**D**) Immunoprecipitation of virions associated to the nuclear fraction. Nuclei isolated after 5 min served as background control. pTyr; phosphotyrosine antibody. qPCR values represent geq/µL.

All the described capsid structural changes, i.e., conformational epitope rearrangement, phosphorylation and DNA externalization, were observed in virions isolated from the cytoplasmic fraction devoid of nucleus. We next analysed the presence of viral DNA and capsids in the nuclear fraction at increasing times pi. The integrity of the isolated nuclei was assessed by light microscopy after trypan blue staining and the purity of the nuclear fraction was verified by the absence of cytoplasmic contamination using antibodies against GAPDH (cytosolic marker), lamin A/C (nuclear inner membrane marker) and SERCA2 ATPase (endoplasmic reticulum marker) (Figure 6A). Nuclei isolated after 5 min or from cells treated with BafA_1_ to prevent endosomal escape served as negative controls. Viral DNA was quantified from the purified nuclei by qPCR and the results were normalized by the quantification of the β-actin gene. An increasing amount of viral DNA accumulated in the nuclear fraction reaching a plateau by 2–3 h pi. In contrast, nuclear viral DNA did not increase significantly above the background level in cells treated with BafA_1_ (Figure 6B).

The accessibility of the viral DNA accumulating in the nuclear fraction was examined by nuclease digestion. Purified nuclei were prepared at 5 min (negative control) and 3 h pi. While the DNA detected 5 min pi, representing the background, was resistant to nucleases, the viral DNA accumulated in the nuclear fraction at 3 h pi was mostly nuclease sensitive. Incubation of the nuclear fraction at 60 °C for 10 min was used to control the activity of DNase I (Figure 6C). 

We next analysed whether the nuclear viral DNA signal originated from free or capsid-associated DNA. To this end, nuclear fractions were prepared at 5 min (negative control) and 3 h pi and used for immunoprecipitation with the Caps, PLA2 and phosphotyrosine antibody. The immunoprecipitated viruses were quantified by qPCR, as specified above. Additionally, the accessibility of the viral DNA was examined by treatments with DNase I. The results showed that only the capsids that underwent the structural modifications in the cytoplasm, i.e., conformational epitope rearrangement, phosphorylation and DNA externalization, were able to reach the nucleus (Figure 6D).

### 3.7. Nuclear Targeting of B19V Is Mediated by the Microtubule-Dependent, Minus-End-Directed Motor Dynein

**Figure 7:**
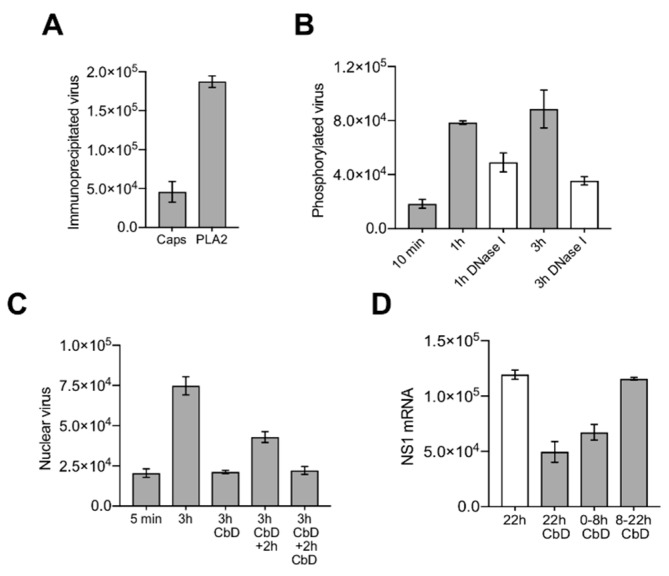
Inhibition of dynein-mediated nuclear targeting blocks the incoming viruses in the cytoplasm but does not prevent the capsid rearrangement, phosphorylation and uncoating (**A**) Effect of the dynein inhibitor ciliobrevin D (CbD; 100 µM) on conformational epitope integrity (Caps/PLA2 ratio). (**B**) Effect of CbD on phosphorylation and DNA externalization. (**C**) Effect of CbD on the nuclear accumulation of B19V DNA. After 5 min (background control) and 3 h pi, cells were washed and the amount of viral DNA was quantified by qPCR. Additionally, cells were incubated for two additional hours in the presence or absence of CbD. All values of qPCR correspond to geq/µL. (**D**) Effect of CbD on B19V infection. Cells were infected in the presence of CbD for the indicated times and the amount of NS1 mRNA was quantified by RT-qPCR at 22 h pi. Values represent NS1 mRNA copies/µL.

Cytoplasmic dynein mediates ATP-dependent retrograde movement of cargoes, including endocytic vesicles, along microtubules toward the centrosome near the nucleus [46]. Accordingly, endocytosed B19V can indirectly benefit from this transport. However, it remains unclear whether parvovirus particles can directly engage motor proteins when they are released in the cytosol for their own transport to the nuclear vicinity. In order to address this question, ciliobrevin D (CbD), which is a specific inhibitor of AAA+ ATPase motor cytoplasmic dynein, was used. CbD inhibits dynein function without altering microtubule structure and dynamics [47,48]. In order to not disturb dynein-mediated endocytic transport of B19V and release from endosomes, cells were treated with CbD 15 min after internalization, at a time when most of the intracellular viruses are in late endosomes [9]. Under these experimental conditions, the loss of the conformational epitope (Caps/PLA2 ratio), capsid phosphorylation and DNA externalization were not disturbed by CbD (Figure 7A,B). In contrast, the accumulation of viruses in the nuclear fraction was prevented. Release of the reversible CbD block resulted in an increased nuclear accumulation of viruses (Figure 7C). As expected, disruption of dynein function by CbD impaired B19V infection, but only when present early during virus entry and not at later times pi (Figure 7D).

### 3.8. Capsids Immunoprecipitated from Cytoplasmic and from Nuclear Fractions Support Complementary-Strand DNA Synthesis

**Figure 8:**
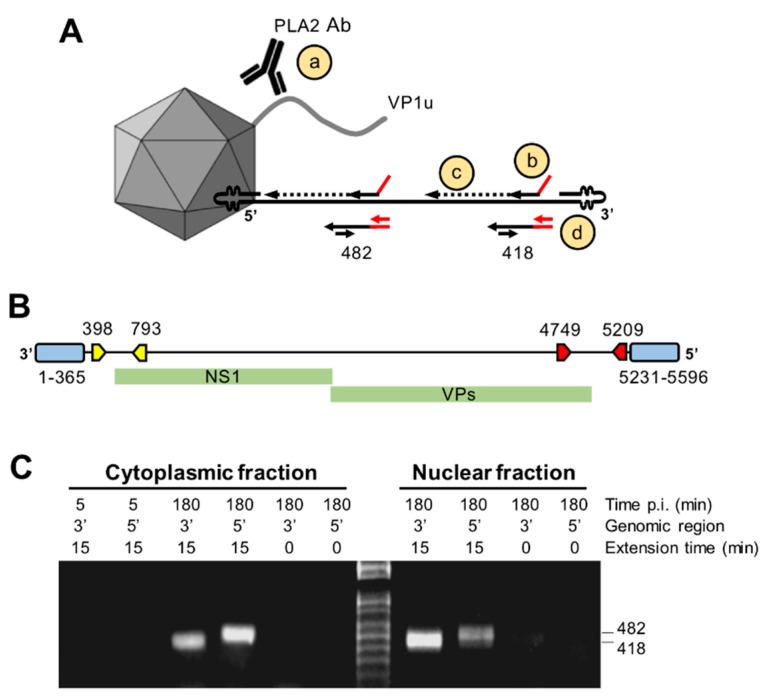
Complementary-strand DNA synthesis from cytoplasmic or nuclear capsids. (**A**) Depiction of the strategy for complementary-strand synthesis: (**a**) Immunoprecipitation of B19V capsids with the PLA2 antibody; (**b**) 5’ virus-unrelated sequence; (**c**) extension reaction by T7 polymerase at 37 °C; and (**d**) amplification of the primer-extended DNA by PCR. (**B**) Positions in the B19V genome of the 3’ (yellow) and 5’ (red) regions targeted by the hybridization and extension assay. (**C**) Viral particles were immunoprecipitated from cytoplasmic or from nuclear fractions with the antibody against the PLA_2_ region (PLA2) and used for complementary-strand DNA synthesis. The primer-extended DNA was amplified by PCR and amplicons of the expected size were visualized by agarose gel electrophoresis. As controls, viruses were immunoprecipitated 5 min pi or incubated with the T7 polymerase for only a few seconds.

The strategy for complementary-strand synthesis is outlined in Figure 8A. Cytoplasmic and nuclear capsids were immunoprecipitated with the antibody against the PLA_2_ region, which remains accessible during cell entry. The presence of externalized DNA in the immunoprecipitated capsids and its suitability as template for complementary-strand synthesis were examined by hybridization with primers targeting the 3’ or the 5’ region of the viral genome (outlined in Figure 8B). The oligonucleotides consisted of a 3’ virus-specific sequence (specific for a sequence stretch at the 3’ or 5’ regions) and a 5’ virus–unrelated sequence. Following the hybridization reaction at 37 °C, the extension was performed at 37 °C for 15 min. Negative controls consisted of viruses immunoprecipitated at 5 min pi or viruses immunoprecipitated at 3 h pi without the polymerase extension step. The reaction was stopped, and the primer-extended DNA was amplified by real-time PCR using a forward primer specific for the 5’ virus-unrelated sequences of the oligonucleotide and a virus-specific reverse primer. Only capsids immunoprecipitated from cytoplasmic fractions or from nuclear fractions at 3 h pi originated the expected PCR product, indicating the presence of accessible DNA templates from the immunoprecipitated capsids. Similar results were obtained by using primers targeting the 3’ or the 5’ region of the viral genome (Figure 8C). DNA accessibility was not detected from capsids immunoprecipitated after 5 min of internalization or in samples without polymerase extension. These results indicate that the viral DNA becomes accessible already in the cytoplasm prior to nuclear entry and supports complementary-strand synthesis while remaining associated to the assembled particle.

## 4. Discussion

Parvovirus infection critically depends on the processing of the incoming particles by cellular factors to promote their transport into the nucleus and the delivery of the viral genome for replication. How and where human parvovirus B19 (B19V) releases the viral DNA and which capsid rearrangement are required for the process is currently unknown. In this study, we combined in vitro and in vivo experiments to address the mechanism of uncoating of B19V.

A common parvovirus structural feature is the combination of antiparallel β-hairpins at the five-fold vertex enclosing a cylindrical structure that connects with the interior of the capsid [35,49,50,51,52]. Structural and in vitro studies suggest that these channels serve as portals for the externalization of N-terminal capsid protein sequences required during the process of virus entry and for the packaging and release of the viral genome [23,31,32,33,34,52,53,54]. Mutations that perturb the structure of the channel result in defective genome encapsidation, uncoating and VP1u externalization [30,31,39]. Those mutational studies have also confirmed that MVM cylinders can mediate progressive 3′-to-5′ genome release, suggesting that this is the strategy of uncoating, and that unknown cellular trigger(s) may initiate the DNA rearrangement during virus entry [39]. In B19V, the channel does not mediate the externalization of N-VP2 sequences, which are already exposed [55], and N-VP1 might also occupy a surface position not accessible to antibodies until the interaction of the virion with cellular receptors [14,15]. Accordingly, the main function of the channel in B19V would be the packaging and release of the viral genome. However, different to other parvoviruses, the channel in B19 VP2 capsids is constricted at its outside end, although three consecutive glycine residues at this site may provide enough flexibility to open the channel upon specific cellular cues [35]. 

Iodixanol density gradient ultracentrifugation followed by qPCR detection of the viral DNA proved to be a useful approach to detect and quantify structural rearrangements in the viral capsid. The method allowed to explore conditions triggering the uncoating reaction at physiological temperatures and to identify and characterize uncoating intermediates. Native capsids in human plasma or in MEM were stable and remained intact after exposure to 37 °C or to a freeze/thaw cycle. However, in the absence of divalent cations or in the presence of chelating activity at 4 °C, the viral capsids rearranged resulting in particles with a lower density and mostly resistant to nuclease digestion. These capsids however were rather unstable and incubation at 37 °C or a freeze/thaw cycle was sufficient to trigger viral DNA externalization without capsid disassembly. These rearrangements were prevented in the presence of 1 mM MgCl_2_ or 1 mM CaCl_2_, strongly suggesting that divalent cations have a stabilizing effect on the viral particle and their removal facilitates the uncoating reaction at physiological temperatures. The TEM images confirmed the presence of assembled particles with variable degree of DNA externalization (Figure 3E), explaining their broader iodixanol density profile (Figure 2). Similar results were obtained with minute virus of mice (MVM). Depletion of capsid-associated divalent cations in MVM rendered the virions unstable and exposure to 37 °C triggered the externalization of the genome, in a 3′-to-5′ direction, leaving the 5′ end of the DNA associated with the capsid. The process was not observed in the presence of divalent cations or in capsids with shorter genomes [42].

During packaging, the viral DNA is loaded at a high density into the small inner cavity of the pre-formed capsids in a 3’ to 5’ direction through the fivefold cylinder [31,33,56,57]. It is conceivable, that in order to condensate and stabilize the DNA-filled capsid, divalent cations may be incorporated into the B19 progeny particle during packaging to neutralize the negative charges of closely packed DNA phosphate backbones and eventually also negative charges in the particle lattice interior. The highly condensed DNA generates a considerable internal pressure and loss of capsid-associated divalent cations during entry may increase the internal tension and destabilize the whole structure, provoking the externalization of the viral genome through the fivefold channel without the need to disassemble the capsid. A direct correlation between the amount of encapsidated DNA and the sensitivity to chelating agents was demonstrated for a variety of of viruses [58,59], including different rAAV vectors and MVM [37,40,42]. Divalent cations may also bind the capsid to cement the structure, as it has been demonstrated for other viruses, and their removal was shown to destabilize the capsid and promote uncoating [60,61,62,63,64]. Prediction of metal ions binding sites by IonCom, which combines the ab initio model with multiple threading alignments [65], revealed a Mg^2+^ and Ca^2+^ binding site in close proximity on the surface of B19V capsid. While the Mg^2+^ binding domain was slightly buried, the Ca^2+^ binding site appeared on the surface surrounding the dimple-like depression at the icosahedral two-fold axis of symmetry (data not shown). 

The similar response of MVM and B19V to divalent cation depletion may reflect a common uncoating strategy of parvoviruses based on a mechanism of DNA externalization without capsid disassembly. This strategy would allow the exceptionally stable parvoviral particles to deliver the genome inside the host cells, and since the particle and the viral DNA remain associated, the capsid can transport the externalized genome to the precise nuclear location to initiate replication and transcription. The destabilization and uncoating of B19 virions under divalent cation depletion in vitro suggests a mechanism for viral genome release in low-divalent-cation environments, such as those typically encountered within the cytosol of the cell, where the activities of ATP-driven Ca^2+^ pumps and Na^+^/Ca^2+^ exchangers maintain the concentration of Ca^2+^ ions at a very low (10–100 nM) level [66]. Therefore, following endosomal escape, the virion is abruptly exposed to a divalent cation-depleted environment in the cytosol, which may destabilize the incoming particle. However, although the in vitro studies reveal important information on conditions allowing the uncoating reaction and allow the characterization of uncoating intermediates, these studies do not necessarily reflect the uncoating process in vivo, which may differ in its mechanism and be triggered by intracellular factor(s) other than divalent cation depletion. With the aim to get insights into the intracellular capsid processing required for B19V uncoating in vivo, we followed capsid modifications and changes in epitope and DNA accessibility during B19V entry. These rearrangements were examined in UT7/Epo cells at various times pi from cytoplasmic and nuclear fractions and under different conditions restraining endosome and dynein functions. 

The first detectable capsid rearrangement during B19V entry was the change in VP1u configuration occurring at the plasma membrane before virus internalization. For most parvoviruses, VP1u externalization occurs inside the endosomes triggered by the acidic environment [11,23,67,68,69]. However, we have previously shown that B19V operates differently. Upon interactions on the surface of susceptible cells, originally inaccessible VP1u sequences become exposed [13] and interact with as yet unknown highly restricted receptor required for virus uptake [14,15]. As expected, cell-bound virions were immunoprecipitated with the antibody against VP1u (PLA2) and the conformational antibody detecting exclusively intact capsids (Caps) (Figure 4C).

The second capsid rearrangement detected was more complex and occurred after endosomal escape in the cytoplasm. While the capsids remained detectable by the VP1u antibody, approximately half of them became undetectable by the conformational antibody against capsids (Figure 4C,D). The capsids that rearranged became sensitive to nuclease digestion in contrast to those that had not rearranged (Figure 4E). The conformational epitope expands three neighboring VP2 molecules related by a five-fold axis and by a three-fold axis [43], and its loss can be explained by a rearrangement occurring at this site. Alternatively, epitope masking by phosphorylation might prohibit antibody binding. In line with this assumption, approximately the same number of incoming capsids that rearranged became phosphorylated (Figure 5C), a process that can only occur after endosomal escape. As expected, endosomal alkalization by bafilomycin A1 (BafA_1_) abrogated endosomal escape and all the capsid rearrangements, including the phosphorylation (Figure 5A,B,E). Similar to BafA_1_, CQ also raises the endosomal pH but, in contrast to BafA_1_, CQ promotes B19V endosomal escape by endosome swelling and rupture (Figure 5A) [9]. Accordingly, alkalization of endosomes by CQ had no effect on B19V capsid rearrangement and phosphorylation (Figure 5B,E). 

Incoming B19V accumulated progressively in the nuclear fraction reaching a plateau by 2 h pi. As expected, blocking endosomal escape by BafA_1_ prevented the nuclear accumulation of the capsids (Figure 6B). While the capsids present in the cytoplasmic fraction represented a mixture of intact and rearranged particles, the capsids associated to the nuclear fraction were mostly those that had rearranged, i.e., phosphorylated, undetectable by the conformational antibody and nuclease sensitive, suggesting the existence of a selective transport towards the nucleus for the capsids with accessible genomes. The accumulation of B19V capsids in the nuclear fraction was reversibly abrogated by ciliobrevin D (CbD), which is a specific inhibitor of the ATPase activity of cytoplasmic dynein [47]. CbD had no effect on the capsid rearrangements in the cytoplasm, including phosphorylation and genome externalization. The interference of dynein function had a significant inhibitory effect on the infection, but only when the reversible drug was present during the first hours of the infection (Figure 7), indicating that the accumulation of the incoming capsids with accessible genomes in the nuclear fraction is required for the infection. At present, it is uncertain how many of these particles are inside the nucleus and how many remain at the cytosolic side. 

The primer hybridization and extension assay confirmed that incoming B19V particles expose the genome already in the cytoplasm prior to nuclear entry, and that the accessible DNA was an optimal template for complementary-strand synthesis (Figure 8). This observation suggests that initiation of DNA synthesis can proceed while the exposed genome remains associated to its capsid.

This study provides a better understanding of the conditions triggering B19V uncoating at physiological temperatures and allowed the identification of capsid structural transitions that precede genome release. Whether incoming viruses are destabilized by the low-cation environment of the cytosol, as suggested by the in vitro data or by another cellular cue, will have to be confirmed. However, our in vivo data reveal that during the process of entry, B19V employs a mechanism of DNA externalization following a limited capsid rearrangement in the cytoplasm to make the genome sufficiently accessible to the replication machinery of the cell.
